# Chemotherapy and the Skin: Understanding Dermatologic Side Effects

**DOI:** 10.7759/cureus.103847

**Published:** 2026-02-18

**Authors:** Pooja Unnikrishnan, Kirankanth Vudayana, Sanjana Diddi, Usha Sri Akkineni, Vunnava Sri Koulini, Chakka Gayathri

**Affiliations:** 1 Dermatology, Venereology and Leprosy, Great Eastern Medical School and Hospital, Srikakulam, IND

**Keywords:** antimetabolite-induced toxicity, chemotherapy, cutaneous adverse drug reactions, hand–foot syndrome, maculopapular eruption, taxane-induced reactions

## Abstract

Background

Chemotherapy-induced cutaneous adverse drug reactions (cADRs) represent a frequent and clinically significant component of treatment-related toxicity in oncology. These reactions may range from mild, self-limiting eruptions to more severe presentations capable of compromising patient comfort, adherence to therapy, and overall quality of life. Their clinical heterogeneity reflects variations in drug class, cumulative exposure, and individual susceptibility. Given the increasing use of combination regimens and targeted therapies, systematic characterization of cADRs remains essential for optimizing supportive care and minimizing treatment interruptions.

Materials and methods

This prospective observational descriptive case series (referral-based cohort) study, which included 30 patients who developed cADRs following administration of single-agent or combination chemotherapy and were subsequently referred from the oncology ward to the dermatology outpatient department (OPD) at a tertiary care center over a one-year period. Demographic, clinical, and treatment-related variables were documented, including latency, morphology, distribution, and severity grading using the Common Terminology Criteria for Adverse Events (CTCAE) version 5.0. Skin biopsies were performed when clinically indicated. Management strategies and clinical outcomes were recorded. Descriptive statistics were used to summarize the data.

Results

Breast carcinoma (33.3%) and gastrointestinal (GI) cancers (23.3%) were the most common underlying malignancies. Taxanes (26.6%), platinum compounds (23.3%), and antimetabolites (20%) accounted for the majority of implicated agents. Predominant cADRs included hand-foot syndrome (HFS) (20%), maculopapular eruptions (16.6%), alopecia (16.6%), nail changes (13.3%), infusion-related urticaria (10%), and pigmentary alterations (6.6%). Most reactions were classified as CTCAE Grade I or II, with only three patients (10%) demonstrating Grade III toxicity. Supportive dermatologic care resulted in favorable outcomes for the majority, and although 20% required temporary dose modification, no patient required permanent cessation of chemotherapy.

Conclusion

Chemotherapy-induced cADRs encompass a broad clinical spectrum, with most reactions being mild to moderate and amenable to timely supportive interventions. Recognition of drug-specific cutaneous patterns and early dermatologic involvement can reduce morbidity, support treatment continuity, and enhance patient satisfaction. Strengthening collaborative care pathways between oncology and dermatology serves an important role in improving overall patient outcomes during systemic cancer therapy.

## Introduction

Cutaneous adverse drug reactions (cADRs) are frequently encountered in patients undergoing systemic chemotherapy and constitute an important component of treatment-related morbidity. Chemotherapeutic agents exert cytotoxic, inflammatory, and immunomodulatory effects on the skin, hair, nails, and mucous membranes, making these tissues particularly vulnerable to therapy-induced injury [1,2]. Dermatologic toxicity may arise from direct eccrine or follicular damage, disruption of keratinocyte proliferation, microvascular injury, or drug-specific molecular pathway inhibition, resulting in a wide spectrum of clinical presentations [3-5]. With the growing use of complex multidrug regimens in oncology, dermatologists increasingly evaluate referred patients with suspected cADRs to facilitate diagnosis, grading, and supportive care.

Taxanes, platinum-based compounds, antimetabolites, anthracyclines, and targeted therapies represent some of the most commonly implicated drug classes in chemotherapy-related cutaneous toxicity [6-10]. Each class exhibits a characteristic pattern of dermatologic manifestations: antimetabolites frequently induce hand-foot syndrome (HFS), erythema, and dysesthesia [11,12]; taxanes are associated with alopecia, onycholysis, nail hyperpigmentation, and infusion-related reactions [13-15]; while platinum compounds and anthracyclines have been linked to pigmentary changes, serpentine supravenous hyperpigmentation, and mucocutaneous irritation [16,17]. Targeted agents, including epidermal growth factor receptor (EGFR) inhibitors and small-molecule kinase inhibitors, contribute additional mechanisms of cutaneous injury and may present with acneiform eruptions, xerosis, or paronychia [18,19].

Although many cADRs are mild and self-limiting, moderate-to-severe reactions may compromise quality of life, increase psychological stress, and precipitate chemotherapy dose delays or modifications [20-22]. Early dermatologic assessment allows accurate differentiation between benign, expected toxicities and potentially serious reactions requiring intervention. The absence of timely evaluation may lead to misdiagnosis, inappropriate treatment cessation, or unnecessary investigations, particularly in oncology patients who often present with overlapping systemic symptoms [23]. Despite the recognized burden of cADRs globally, regional patterns vary depending on chemotherapy protocols, demographic factors, and institutional treatment practices. Limited data exist from Indian tertiary centers describing real-world dermatology referrals from oncology services. Understanding these patterns is essential for refining supportive care strategies, optimizing treatment adherence, and improving overall patient outcomes [24,25].

Accordingly, this study aimed to systematically document the epidemiology, morphology, severity, and management outcomes of chemotherapy-induced cADRs in 30 patients referred from the oncology ward to the dermatology outpatient department (OPD) of a tertiary care cancer center. This study aimed to describe the epidemiology, morphology, severity grading (Common Terminology Criteria for Adverse Events, version 5.0 or CTCAE v5.0), and management outcomes of chemotherapy-associated cutaneous adverse drug reactions (cADRs) referred from oncology services to dermatology at our center.

## Materials and methods

Study design

This single-center prospective observational descriptive case series (referral-based cohort) study was conducted from March 2024 to March 2025 in the Dermatology Outpatient Department (OPD) of Great Eastern Medical School and Hospital, Srikakulam, Andhra Pradesh, India. The study evaluated patients referred from the oncology ward with suspected chemotherapy-induced cutaneous adverse drug reactions (cADRs). Ethical approval for the study was obtained from the Institutional Ethics Committee on March 20, 2025 (Reg. No. 10/IEC/GEMS&H/2025). This is a referral-based descriptive case series (n=30) intended to describe patterns rather than provide population-level generalizability.

Study participants

The study population included patients of all ages and both sexes who were undergoing single-agent or combination chemotherapy for histologically confirmed malignancies and subsequently developed new-onset dermatologic symptoms temporally related to chemotherapy. Only cases referred from the oncology ward to the dermatology OPD for evaluation were included. Patients with incomplete documentation, those receiving radiotherapy alone, those with pre-existing uncontrolled skin diseases, or those whose reactions were attributable to non-oncology medications were excluded. A total of 30 patients fulfilled the clinical and temporal criteria for chemotherapy-induced cADRs and were included in the analysis.

Data collection

For each patient, detailed demographic information, oncologic diagnosis, chemotherapy regimen, cumulative exposure, and latency of onset of dermatologic symptoms were recorded from clinical files. Dermatologic assessments conducted at the time of referral included evaluation of morphology, distribution, mucosal involvement, appendageal changes, and symptom severity. Clinical photographs were obtained with patient consent. Laboratory investigations and skin biopsies were performed only when clinically indicated to confirm diagnosis or exclude mimickers. All data were extracted from dermatology and oncology case records and compiled systematically for analysis. Immune checkpoint inhibitors were not represented in this cohort, as no eligible ICI-exposed referrals were captured during the study period. Chemotherapy exposure included both single-agent and combination regimens. Of the 30 patients included, 12 patients (40%) received single-agent chemotherapy, while 18 patients (60%) were treated with combination regimens. In patients receiving combination therapy, attribution of cutaneous adverse drug reactions was performed conservatively based on temporal relationship to drug administration, known toxicity profiles of individual agents, and documentation provided by the treating oncologist. This limitation was acknowledged during analysis, as combination regimens may confound precise causality assessment of individual agents.

Severity assessment

The severity of cADRs was graded using CTCAE v5.0, which classifies adverse events from Grade I (mild) to Grade V (death). Grading incorporated the extent of cutaneous involvement, presence of functional impairment, symptom burden such as pain or burning, and any mucosal or appendageal involvement. Two dermatologists independently assessed each case, and discrepancies were resolved by consensus.

Statistical analysis

All data were entered into Microsoft Excel (Microsoft® Corp., Redmond, WA) and analyzed using the OpenEpi Version 3.01 software (Emory University, Atlanta, GA). Descriptive statistics were used to summarize demographic characteristics, malignancy distribution, clinical patterns, reaction severity, and treatment responses. Associations between selected categorical variables were examined using Chi-square tests, with statistical significance defined as a p-value < 0.05.

## Results

**Figure 1:**
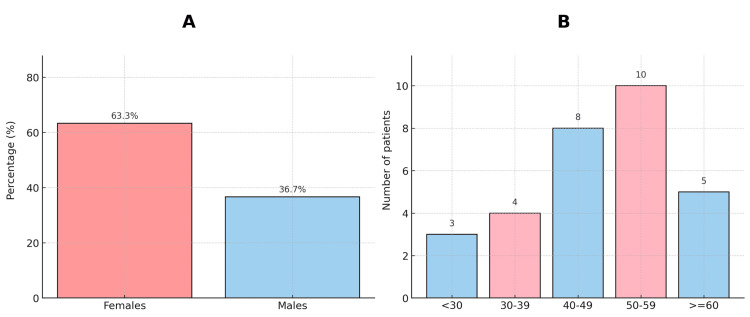
Bar chart illustration showing age and gender distribution (A) Age distribution, (B) Gender distribution

A total of 30 patients referred from the oncology ward to the dermatology outpatient department were included in the study. The mean age was 51.4 years (range: 22-72), with a female predominance (63.3%) (Figures 1A, 1B). Breast carcinoma was the most common underlying malignancy (33.3%), followed by gastrointestinal and hematological cancers (23.3%) (Table 1).

**Table 1:** Distribution of underlying malignancies (N = 30)

Malignancy	Number (n)	Percentage (%)
Breast carcinoma	10	33.3
Gastrointestinal malignancies	7	23.3
Other malignancies (including hematological cancers)	13	43.4
Total	30	100

Among the study population, 12 patients (40%) were receiving single-agent chemotherapy, whereas 18 patients (60%) were on combination regimens. Cutaneous adverse drug reactions occurred in both groups, with no statistically significant difference in severity between single-agent and combination therapy recipients (p > 0.05). However, causality attribution was more challenging in combination regimens, necessitating conservative interpretation based on clinical timing and established drug-specific toxicity patterns. Taxanes, platinum agents, and antimetabolites were the most frequently implicated drug classes (Table 2). Clinical presentations varied widely, with hand-foot syndrome (HFS), maculopapular eruptions, alopecia, and nail changes forming the most common patterns. Most reactions occurred after the second or third chemotherapy cycle and were classified as Grade I or II according to CTCAE v5.0. Only three patients developed Grade III reactions, and none required permanent discontinuation of chemotherapy.

**Table 2:** Chemotherapy agents, clinical patterns, and severity of cutaneous adverse drug reactions (N = 30) CTCAE v5.0: Common Terminology Criteria for Adverse Events, version 5.0.

Category	Subcategory	Number (%)
Chemotherapy agents	Taxanes	8 (26.6%)
	Platinum compounds	7 (23.3%)
	Antimetabolites	6 (20%)
	Anthracyclines	3 (10%)
	Targeted therapies	3 (10%)
	Alkylating agents	2 (6.6%)
	Others	1 (3.3%)
Clinical patterns	Hand–foot syndrome (HFS)	6 (20%)
	Maculopapular eruption	5 (16.6%)
	Alopecia	5 (16.6%)
	Nail changes	4 (13.3%)
	Infusion-related urticaria	3 (10%)
	Serpentine hyperpigmentation	2 (6.6%)
	Ichthyosis	2 (6.6%)
	Photosensitivity/acneiform reactions	3 (10%)
Severity (CTCAE v5.0)	Grade I	16 (53.3%)
	Grade II	11 (36.6%)
	Grade III	3 (10%)
	Grade IV/V	0

The distribution of implicated chemotherapeutic drug classes showed taxanes as the leading cause, followed by platinum-based compounds and antimetabolites. A smaller proportion of reactions occurred with anthracyclines, targeted therapies, and alkylating agents. This distribution closely parallels global trends reported in previous studies and reflects the prevalent chemotherapy regimens administered at our center.

**Figure 2:**
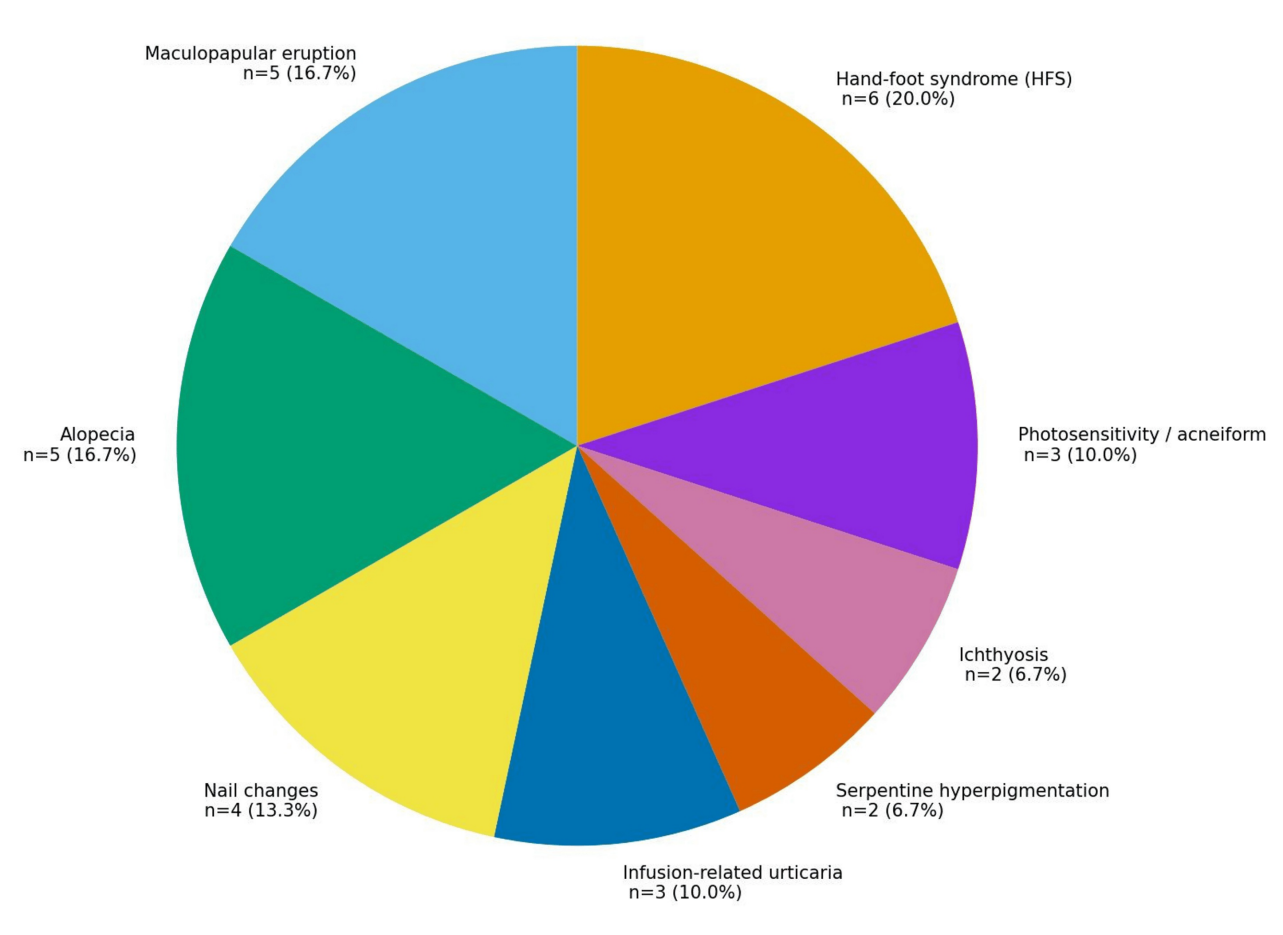
Pie chart showing proportion of cutaneous drug reactions

**Figure 3:**
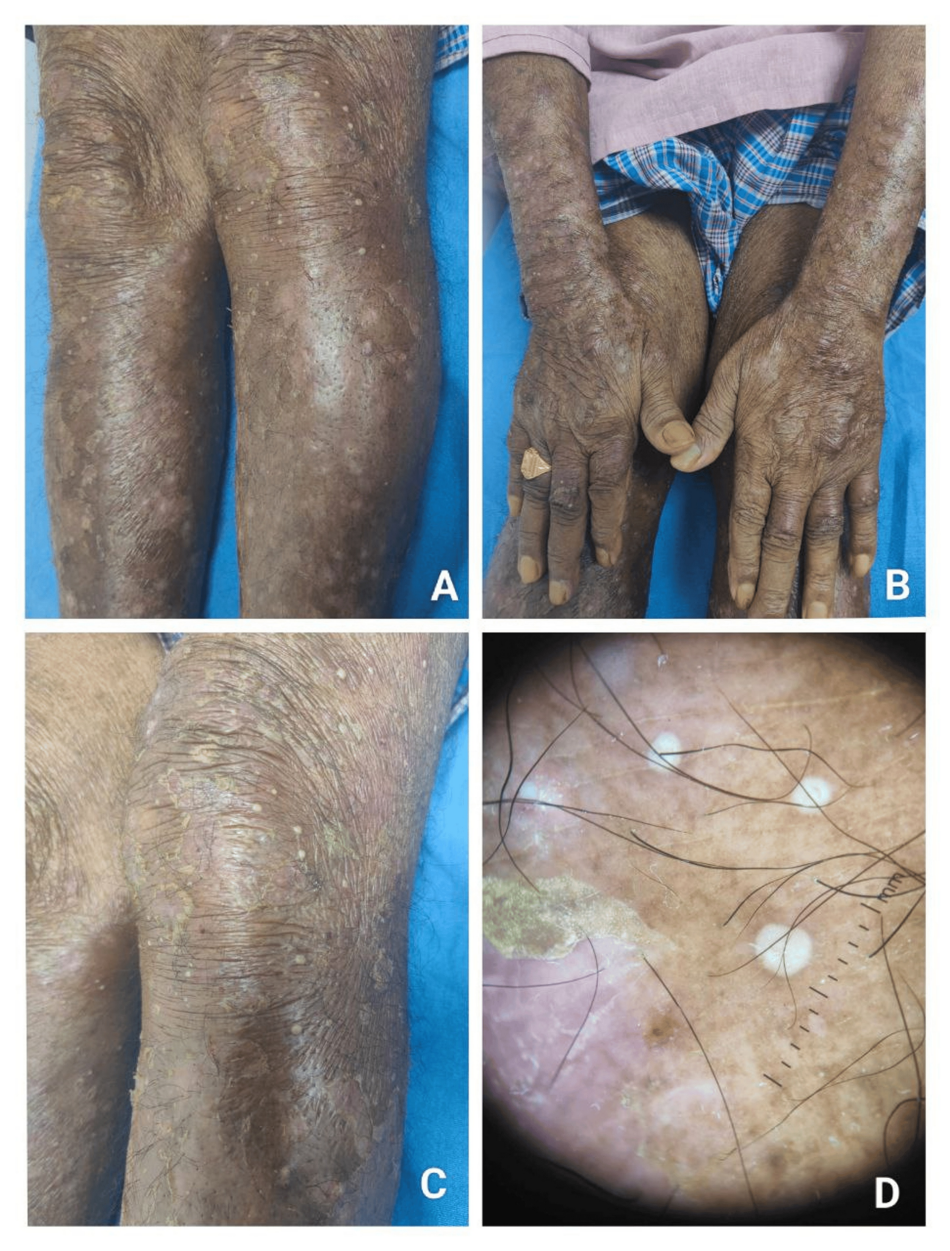
Papulopustular eruption after gefitinib therapy (A) Papulopustular eruptions over extensor aspect of both lower limbs, (B) over dorsum of both hands, (C) Multiple pustular leions over lower limb, (D) Dermoscopic image showing circumscribed yellowish white areas denoting pustular lesions

**Figure 4:**
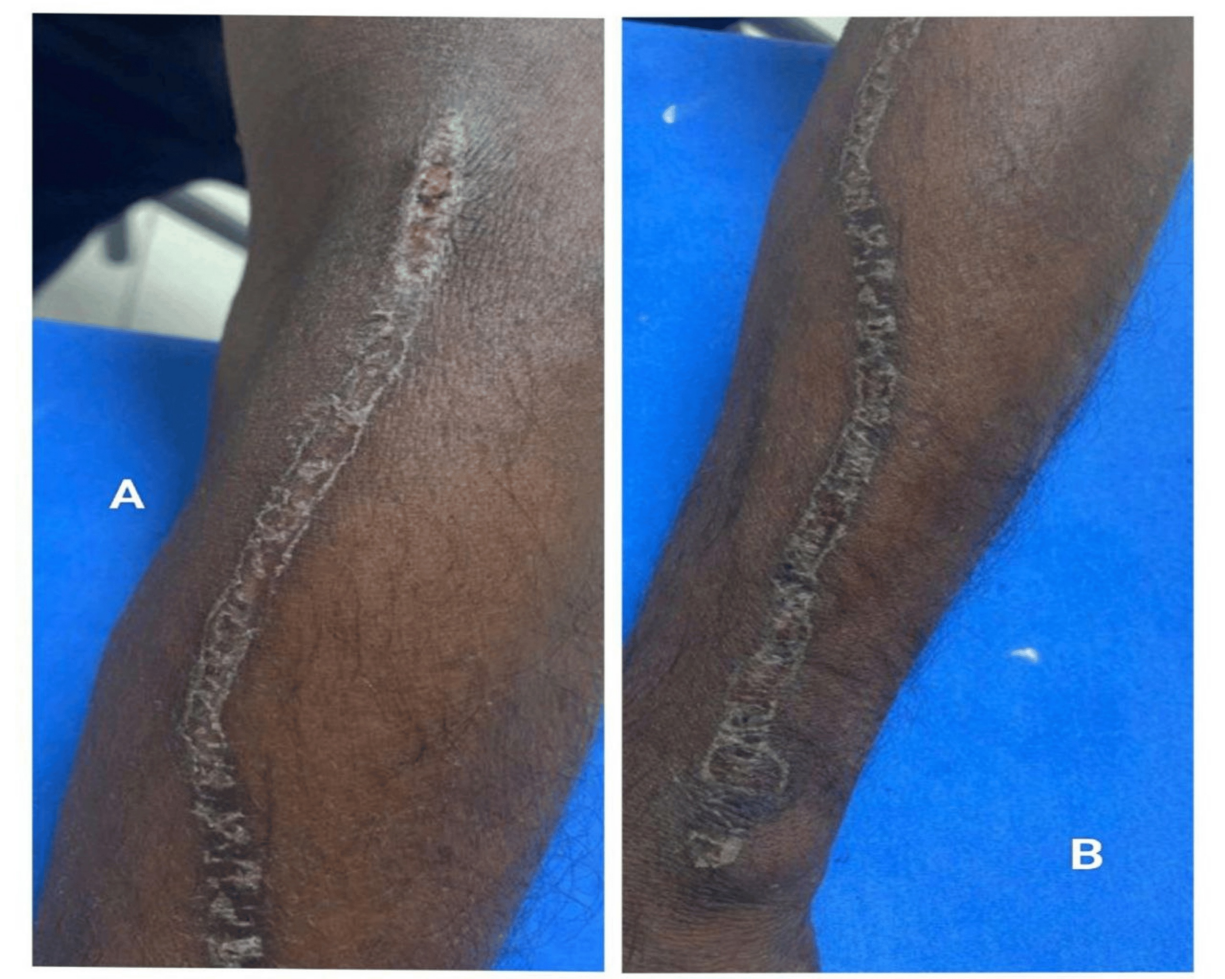
Serpentine supravenous hyperpigmentation secondary to 5-fluorouracil infusion (A) Serpentine supravenous hyperpigmentation developing after 5-fluorouracil infusion, (B) Similar linear hyperpigmented streaks along the venous tract on the opposite limb.

**Figure 5:**
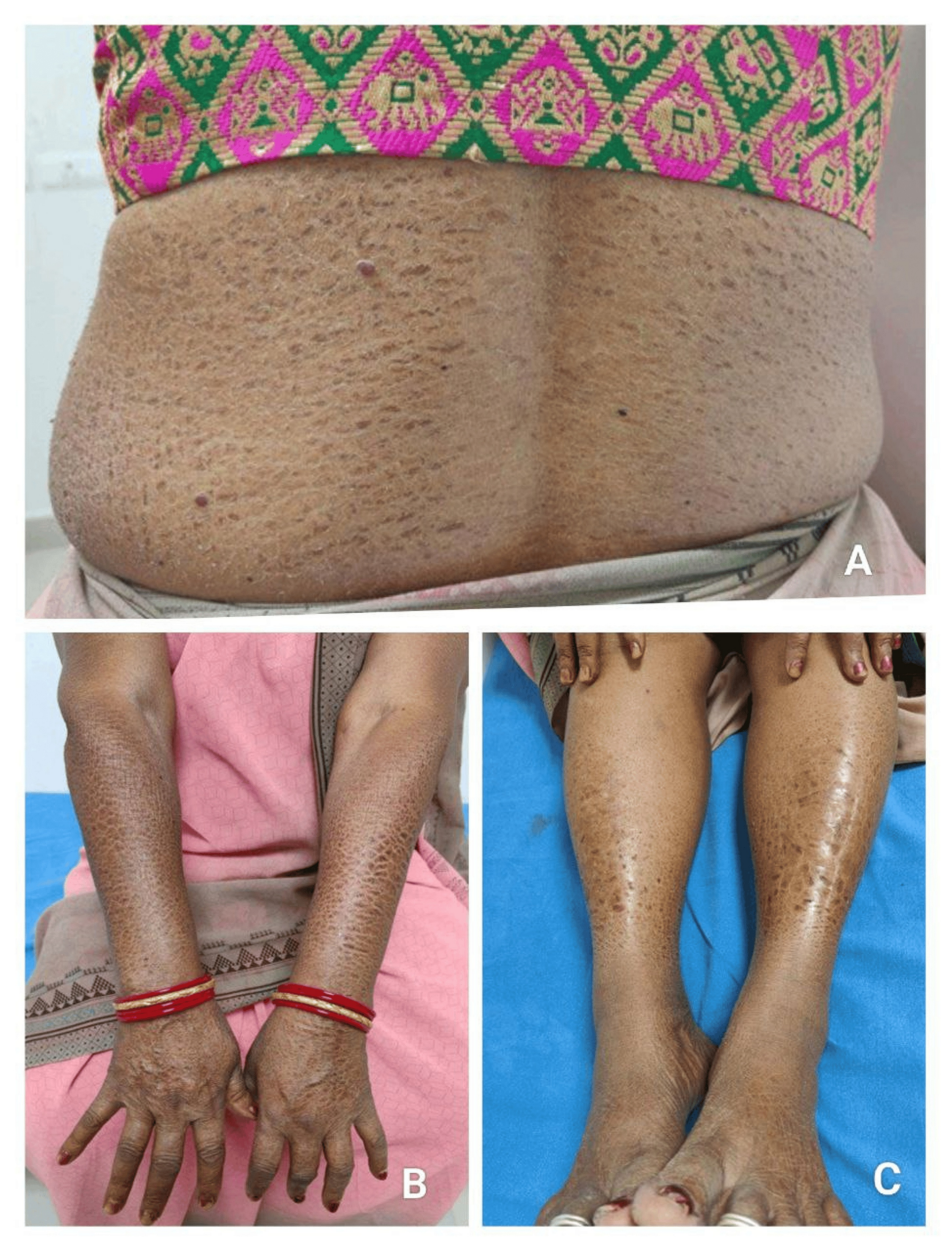
Paclitaxel induced ichthyosis (A) Diffuse ichthyosiform scaling with generalized xerosis over the trunk following paclitaxel chemotherapy, (B) Symmetric ichthyosiform scaling and hyperpigmentation involving the forearms and hands, (C) Prominent ichthyotic scaling over the bilateral lower limbs with accentuation over the shins.

**Figure 6:**
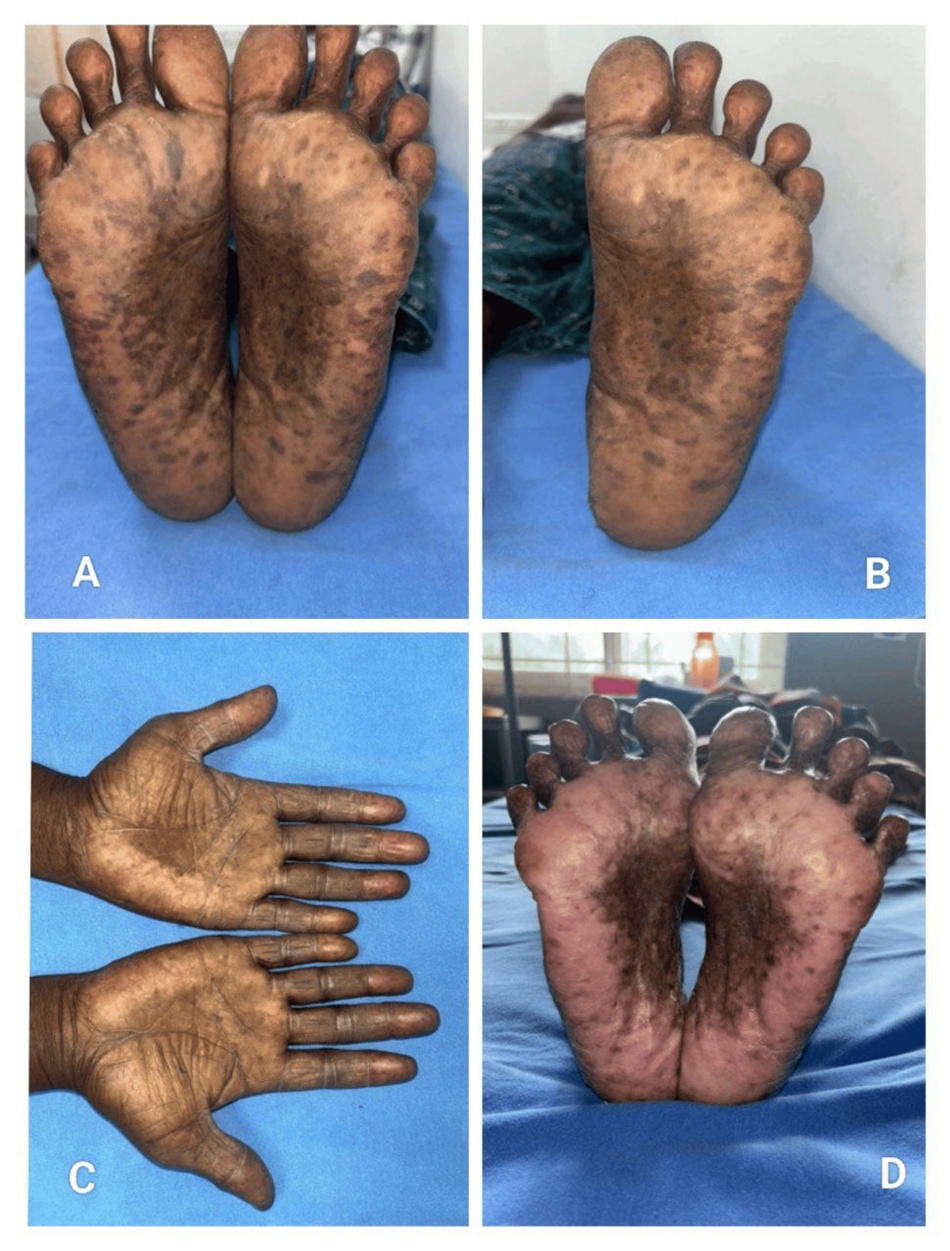
Erythrodysesthesia presented in a patient on capecitabine (A) Bilateral plantar surfaces showing diffuse hyperpigmentation with accentuation over pressure-bearing areas, (B) Close-up view of the sole demonstrating patchy hyperpigmentation and thickening of the plantar skin, (C) Palmar involvement with diffuse hyperpigmentation and textural changes of the hands, (D) Symmetric erythema and hyperpigmentation of the soles, consistent with chemotherapy-induced erythrodysesthesia.

**Figure 7:**
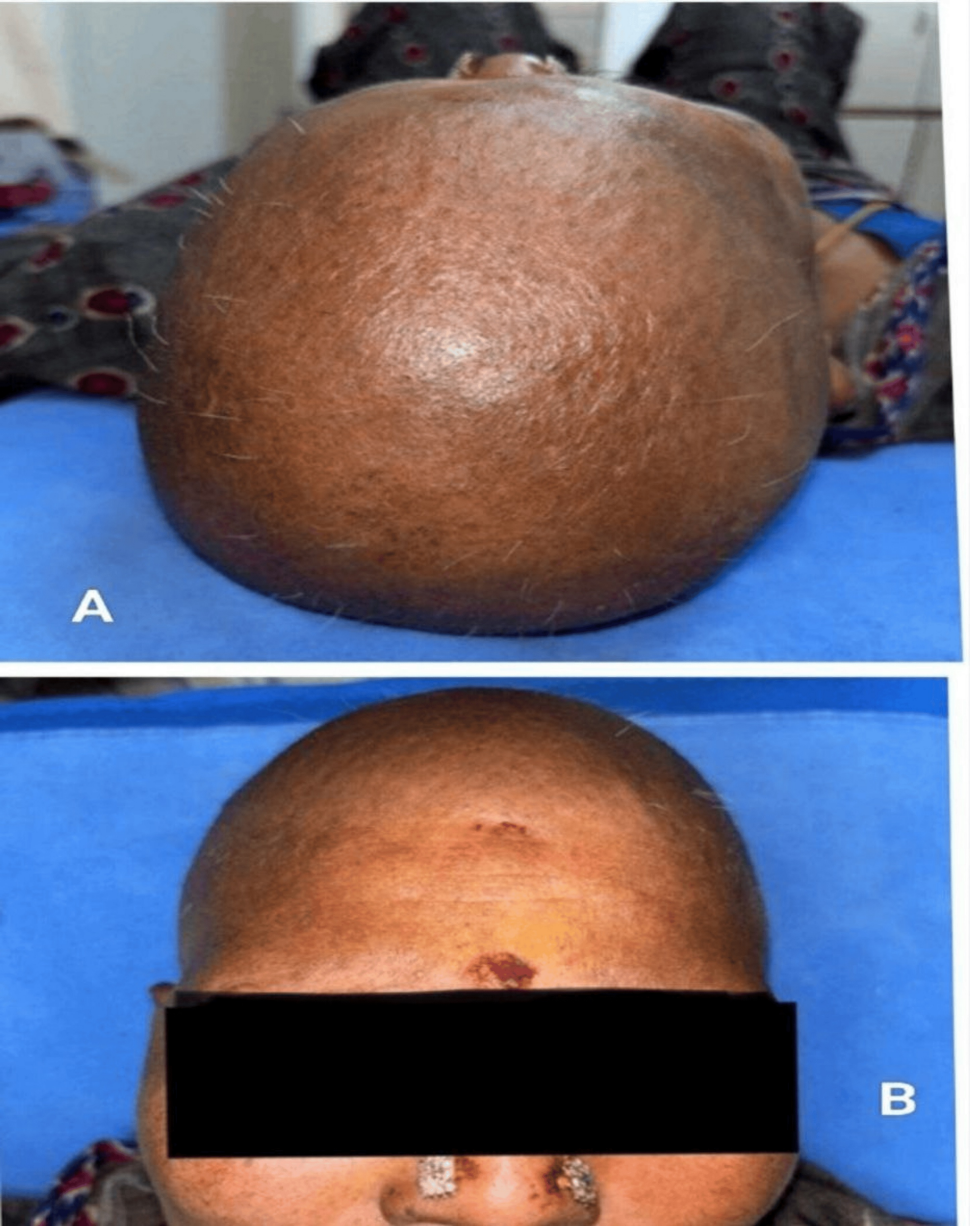
Anagen effluvium after paclitaxel (A) Extensive scalp alopecia due to paclitaxel-induced anagen effluvium, (B) Frontal view demonstrating diffuse anagen hair loss.

Clinical manifestations demonstrated considerable variability. HFS was the most frequent reaction, particularly among patients receiving capecitabine (Figure 2). Maculopapular eruptions occurred commonly with taxanes and platinum agents (Figure 3), while alopecia and nail changes were frequently associated with paclitaxel. Less common patterns included serpentine supravenous hyperpigmentation (Figure 4), ichthyosis (Figure 5), erythrodysesthesia (Figure 6), anagen effluvium (Figure 7), photosensitivity, and infusion-related urticaria. Observed nail changes included onycholysis, nail plate dystrophy/fragility, longitudinal ridging, and melanonychia (nail pigmentation), predominantly seen with taxane-based regimens.

Severity grading demonstrated that more than half of the cohort experienced Grade I reactions, while a smaller proportion experienced Grade II or III severity (Table 2). Importantly, no patients manifested Grade IV or life-threatening reactions. Supportive care, primarily emollients, antihistamines, topical corticosteroids, and symptom-directed therapy, was adequate in the majority. Six patients required temporary dose modification, but none required cessation of oncologic therapy, underscoring the manageable nature of most chemotherapy-induced cADRs.

On statistical analysis, hand-foot syndrome showed a significant association with higher severity (Grade III) reactions (p = 0.04). No statistically significant association was observed between the severity of cutaneous adverse drug reactions and sex, chemotherapy class, or chemotherapy cycle at onset (p > 0.05) (Table 3).

**Table 3:** Association between patient characteristics and severity of cutaneous adverse drug reactions (N = 30) Categorical variables were analyzed using the Chi-square test; Fisher’s exact test was applied where expected cell counts were <5. The Chi-square (χ²) or Fisher’s exact test statistic values are provided in the table. A p-value <0.05 was considered statistically significant.

Variable	Grade I–II (n = 27)	Grade III (n = 3)	Test statistic	*p*-value
Sex			χ² = 0.64	0.42
Male (n = 11)	9	2		
Female (n = 19)	18	1		
Chemotherapy class			χ² = 1.78	0.18
Taxanes (n = 8)	6	2		
Non-taxanes (n = 22)	21	1		
Chemotherapy cycle at onset			Fisher’s exact = 1.02	0.31
≤ 1st cycle (n = 6)	6	0		
≥ 2nd cycle (n = 24)	21	3		
Clinical pattern			Fisher’s exact = 4.23	0.04*
Hand–foot syndrome (n = 6)	4	2		
Other patterns (n = 24)	23	1		

## Discussion

Chemotherapy-related cutaneous adverse drug reactions (cADRs) are well documented in the literature and commonly include hand-foot syndrome, maculopapular eruptions, alopecia, and nail abnormalities, which is consistent with the clinical spectrum observed in our 30-patient series [21,23,24]. Several large reviews emphasize that antimetabolites and taxanes are frequent culprits of HFS and nail toxicities respectively, aligning with our finding that antimetabolites and taxanes accounted for a large proportion of cases [23,24]. Prior work has shown that the majority of chemotherapy skin toxicities are low to moderate in severity and respond to supportive measures without permanent treatment discontinuation, a pattern reflected in our cohort where most reactions were CTCAE Grade I-II and resolved with conservative therapy [23-25].

Nail changes and pigmentary alterations have been described since earlier clinical series and remain an underappreciated source of morbidity; our observations corroborate historical and contemporary reports [21,24]. The temporal relationship between cycle number and onset described by other authors-often after the second or third cycle-was also seen in our patients, supporting predictable latency periods for many drug classes [24,25]. Mechanistic reviews attribute HFS to drug accumulation in eccrine-rich acral skin and to direct cytotoxic effects on keratinocytes, which helps explain the clustering of HFS among capecitabine and 5-fluorouracil recipients in our dataset [22,24]. Taxane-related alopecia and nail dystrophy have been mechanistically linked to mitotic arrest in rapidly dividing follicular and nail matrix cells, a biological basis that concords with our clinical correlations between paclitaxel exposure and these appendageal changes [23,24]. Targeted agents produce distinct patterns (for example, acneiform/papulopustular eruptions with EGFR inhibitors) and require tailored management; although targeted therapies accounted for fewer cases in our study, the phenotypes we observed were consistent with those described in clinical and mechanistic reviews [22,23].

Comparative incidence rates across series vary because of differences in patient populations, chemotherapy regimens, and referral thresholds, yet the relative prominence of HFS, maculopapular rashes, and alopecia in our study mirrors that reported in contemporary oncology dermatology reviews [23,24]. Management algorithms published in recent reviews advocate patient education, emollients, topical corticosteroids, dose-modification guidance, and collaboration with oncology teams; our management approach followed these recommendations and achieved favorable outcomes in most patients [23-25]. Although no patient in our cohort required permanent discontinuation of chemotherapy, six patients required temporary dose modification, indicating that even predominantly mild-to-moderate reactions may influence treatment delivery and require coordinated supportive care. In the present series, most cutaneous adverse drug reactions were mild to moderate in severity and responded favorably to supportive dermatologic care, with only 20% of patients requiring temporary chemotherapy dose modification and none requiring permanent discontinuation. These findings suggest that early dermatologic evaluation facilitates symptom control and supports continuation of planned oncologic therapy. However, given the descriptive nature of this study, definitive conclusions regarding the impact of dermatology intervention on treatment outcomes cannot be established. Nevertheless, our results highlight the practical value of dermatology-oncology collaboration in identifying drug-specific reaction patterns, guiding conservative management strategies, and minimizing unnecessary treatment interruptions in routine clinical practice.

This study has several limitations. The single-center design and modest sample size restrict the generalizability of the findings. As this was a referral-based observational case series, incidence rates may not reflect the broader oncology population. Importantly, immune checkpoint inhibitors (ICIs), which represent a cornerstone of contemporary oncology practice, were not included in this cohort, as no eligible ICI-exposed referrals were captured during the study period. This limits the applicability of our findings to modern immunotherapy-related cutaneous toxicities. In addition, the inclusion of patients receiving combination chemotherapy regimens complicates precise causality attribution to individual agents. The relatively short follow-up period also precludes assessment of delayed or chronic dermatologic toxicities.

## Conclusions

Chemotherapy-induced cutaneous adverse drug reactions are a frequent and clinically important component of treatment-related toxicity in patients receiving systemic anticancer therapy. The common patterns observed included hand-foot syndrome, maculopapular eruptions, alopecia, nail changes, and pigmentary alterations, indicating that chemotherapy can affect multiple cutaneous structures, including the skin, hair, and nails. Most reactions were manageable with supportive therapy such as emollients, topical agents, antihistamines, and symptom-directed measures.

Chemotherapy-induced cutaneous adverse drug reactions represent a diverse clinical spectrum, most of which are mild to moderate in severity and manageable with timely supportive dermatologic care. In this observational series, no patients required permanent discontinuation of chemotherapy due to dermatologic toxicity, although temporary dose modification was necessary in a subset. These findings emphasize the importance of early recognition, appropriate grading, and symptom-directed management of cADRs. While dermatologic consultation appears to aid in symptom control and treatment tolerability, larger prospective studies incorporating contemporary systemic therapies, including immune checkpoint inhibitors, are required to better define the impact of integrated dermatology-oncology care on long-term treatment outcomes.
